# Dermatitis artefacta and psychiatric illness: Brief review and case report

**DOI:** 10.1192/j.eurpsy.2021.1628

**Published:** 2021-08-13

**Authors:** C. Oliveira, F. Caldas, M. Gonçalves

**Affiliations:** Internamento C, Hospital de Magalhães Lemos, Porto, Portugal

**Keywords:** Dermatitis artefacta, factitial dermatitis, psychodermatology, psychocutaneous illness

## Abstract

**Introduction:**

Psychodermatologic disorders are conditions involving an interaction between the mind and the skin. Dermatitis artefacta (DA), also known as factitial dermatitis, is a frequently unrecognized psychocutaneous illness, in which the patient creates skin lesions to satisfy the unconscious need to presume a sick role. It is more common in women and in patients with a diagnosis of psychiatric illness. This is an exclusion diagnosis and organic causes should be ruled out. Treatment of DA can be challenging and it needs to involve a multidisciplinary approach consisting of dermatologists and mental health professionals.

**Objectives:**

From a case report the authors intend to present a literature review of dermatitis artefacta.

**Methods:**

Observation the patient and review the clinical file. Consultation published and referenced scientific articles on PubMed.

**Results:**

60 year old man, diagnosed with Bipolar Disorder, was admitted for manic decompensation of his pathology. During physical examination he had sparse erythematous lesions, more exuberant in the neck, scalp, belly and upper limbs. The diagnosis of artifact dermatitis was made after excluding other possible causes.

**Conclusions:**

Treatment of DA can be challenging and it needs to involve a multidisciplinary approach. Dermatitis artefacta is a long-term disorder, and patients need regular follow up with a dermatologist and a psychiatrist because relapses are common. These doctors must be aware of this possible pathology in order to make a correct diagnosis and treatment of psychiatric disorders that sometimes coexist with skin lesions. The prognosis for most patients is poor leading to self-injury, scarring and poor cosmesis.

**Disclosure:**

No significant relationships.

